# A study on the impact of official promotion short videos on tourists’ destination decision-making in the post-epidemic era

**DOI:** 10.3389/fpsyg.2022.1015869

**Published:** 2022-11-24

**Authors:** Jing Jiang, Yuxin Hong, Wenwen Li, Ding Li

**Affiliations:** ^1^School of Management, Anhui University, Hefeii, China; ^2^School of Public Administration, Southwestern University of Finance and Economics, Chengdu, China

**Keywords:** uncertainties, official short videos, tourism promotion, travel destination decision, SOR theory

## Abstract

The COVID-19 pandemic has had an enormous impact on traditional tourism. Influenced by the uncertainties of the current epidemic, to revive the development of tourism and local economics, local governments have used short video accounts to release tourist promotion short videos for publicizing and marketing. This study takes official promotion short videos as the research object, establishes a structural equation model based on the SOR theory, and explores the influencing factors of official short videos on tourists’ destination decisions through empirical analysis. It finds that the official promotion short videos can positively stimulate users’ perception and sentiments significantly due to the three unique features of authority, interactivity and interest, thus influencing tourists’ destination decision-making behavior. On this basis, this study proposes to improve the operation of an official short video from three aspects: enhancing the authority of official short video accounts, attaching importance to the interactive mechanism, and strengthening the creativity of short videos. It is hoped that the study may help enhance the influence of official promotion short videos and promote the high-quality development of local tourism.

## Introduction

The Covid-19 epidemic spreading all over the world in 2020 not only significantly influences traditional service industries such as catering, accommodation, and tourism, but also changes consumers’ consumption habits and psychology to some extent. Affected by the COVID-19 epidemic, the number of China’s domestic tourists and tourism income both dropped sharply in 2020, and the loss of total tourism revenue was more than $600 billion (Qiang, 2022). Rural tourism is one of the economic activities hardest hit by the pandemic (Eslami and Namdar, 2022). This is undoubtedly disastrous for local governments that rely on tourism to eliminate poverty and develop their economies.

At present, the uncertainties of epidemic development make all industries in China take normalized epidemic prevention and control measures. Therefore, the traditional tourism publicity and promotion mode can no longer match the objectives of normalized epidemic prevention and control policy, and the tourism marketing problem has become prominent increasingly. On the other hand, after long-term home quarantine, residents may have some revenge spending, and there will be a great travel demand during the stable epidemic period. How to promote tourism through a brand-new model at a time of heightened uncertainties and risks and then revitalize the tourism industry and increase tourism revenue has become an urgent problem for local governments to solve.

Technological advances and the use of social media play an important role in driving economic development (Li et al., 2021). After the outbreak of the COVID-19 epidemic, the short video platform has become the main channel not only for the public to obtain information related to the epidemic, but also for the local governments to interact with the public. On the one hand, short videos in marketing reduces consumers’ resistance to marketing and make the products more deeply rooted in people’s hearts (Du and Lee, 2020). On the other hand, with the help of the short video platform accounts, local governments can create an “Internet celebrity city” with huge online traffic, which can attract tourists and promote the development of local tourism industry and the region. For instance, using the short video platforms, DING Zhen, a Tibetan boy from Litang County, Sichuan Province, became popular on the Internet. After his popularity, DING Zhen became the “image spokesperson of Litang” to help his hometown get rid of poverty. With his efforts, Litang County received more than 1,512 million tourists in 2020, achieving a tourism income of 1.66 billion yuan, an increase of 158.1% year on year, reaching a record high.

Live streaming, as a new feature of the short video platform, is of significance to the recovery of tourism (Liu et al., 2022). For example, LIU Hong, director of Sichuan Ganzi Culture and Tourism Bureau, HE Jiaolong, deputy director of Xinjiang Yili Culture and Tourism Bureau, and other local tourism department leaders walked into Douyin short video live streaming room to promote local tourism resources for Internet users with their government official identity.^1^ The government’s promoting tourism resources and publicizing travel destinations through short video platforms has become a new model to attract tourists and promote the development of local tourism under the current background of uncertain epidemic development.

Therefore, this study aims to explore how to better use the official promotion short videos to promote tourism resources and effectively guide potential consumers’ travel consumption under the background of the epidemic uncertainties. Based on the stimulus-organism-response (SOR) theory, this study constructs a structural equation model of the official promotion short videos’ influence on tourists’ destination decision-making behavior. With the online questionnaire data, the relationship between variables was tested by the structural equation model, and then the influencing factors of official promotion short video on tourist destination decision were analyzed. It is hoped that the present study may provide decision-making reference for promoting the development of local tourism.

## Literature review

### Tourist destination image and tourism promotion

The image of a tourist destination refers to the overall impression of a tourist destination interwoven with various tourism products and elements (Huang et al., 2002). In subsequent studies, this impression is further expanded into customer-based brand equity of tourist destinations, which increases the dimensions of perception, quality and loyalty of tourist destinations (Konečnik, 2010). The representative image that a destination tries to communicate with the tourists is called the projected image (Barich and Kotler, 1991), and this is the way a tourist destination gets itself known to more. And the emergence of popular attractions is the result of a synergy between the perceived and predicted images of the destination. Destination Management Organization (DMO), as the main body of image projection, needs to pay attention to the deviation between the official-projected image and the user-perceived image, and conduct management marketing based on the difference (Briciu et al., 2019). Since the projected image of a tourist destination is affected by the tourists’ perceived image, it is necessary to build a good destination image through various promotion and marketing means, change the stereotyped cognition of tourists, and then affect their choice of destination (Kavaratzis and Ashworth, 2008).

### Factors influencing tourist destination decision

Tourists’ destination decision-making behavior is influenced by many factors, including tourists’ internal psychological quality and external social environment (Mayo and Jarvis, 1981). The tourists’ perceived image of the destination before making the decision plays an important role in their decision, so it is necessary to focus on marketing the image of the tourist destination (Baloglu and McCleary, 1999). Under the circumstances of the current global outbreak, the main factor that affects tourists’ destination decision-making is the assessment of the visual attractiveness of the tour (Rogach et al., 2020). Therefore, the promotion and marketing of tourist destinations remain important in the post-pandemic era. More than that, safety should be one of the factors that influence tourists’ decision. Studies have confirmed that travel safety and security (TSS) affect the travel destination image (TDI) in the early decision-making process. Therefore, the destination marketing organization (DMO) should develop appropriate tourism marketing and management strategies to improve destination attractiveness and competitiveness (Hsu et al., 2017). In addition, some scholars in China (e.g., Wang et al., 2007; Guo, 2009) have also found through research that personal perceived value also has an influence on tourists’ destination decisions and purchase intentions.

### Tourism promotion and short video marketing

With the development of new media platforms, short videos have gradually become the main channel of constructing, publicizing, and marketing the image of tourist destinations in recent years. From the perspective of tourist perception, Xiao et al. (2020) found through empirical research that Tik-Tok can affect tourists’ perception of destination image and travel intention, especially that interactive marketing plays a more significant role in the influence of short video marketing on image perception. Cao et al. (2021) also confirmed that short videos can be an effective marketing strategy for tourist destinations. On this basis, the government’s local tourism departments have also gradually strengthened the construction of short video accounts, using the celebrity effect of “Internet celebrities” to stimulate the public’s travel interest and promote the development of local tourism (Zhang and Huang, 2022).

To sum up, short video, an emerging media communication platform, has a significant impact on tourists’ destination choice, and the official-projected image of local destination will also affect tourists’ perception. However, few studies took the official short video account as the research object to discuss its impact on tourist destinations. In the post-epidemic era, China’s tourism industry is also actively seeking for transformation and development and attracting consumers with new ways of travel. The government, as the regulator of the tourism industry, has the responsibility and obligation to promote the high-quality development of tourism while taking epidemic prevention and control measures and creating a safe consumption environment. Driven by the intelligent and digital transformation of the tourism industry, official promotion short videos become the first choice for the government to involve in tourist destination marketing and promotion. Through the planning and operation of official short video account and the shooting and release of official promotion short videos, the government can not only set up a good government image, but also promote the development of local economy, using government affairs new media to boost local tourism industry. Under the background of uncertain epidemic development, it is of great significance to use the official short video account to publicize and market the development of local tourism. Therefore, this study uses empirical research methods to analyze the influencing factors of official short videos on tourists’ destination decision-making. It is hoped that this study may provide a reference for local governments to use short video platforms efficiently to attract tourists, publicize tourism resources, and promote the development of local tourism.

## Theoretical framework and research design

### Theoretical framework

Mehrabian and Russell (1974) proposed the famous stimulation-response model on the basis of environmental psychology, which pointed out that the external environment has an impact on the cognitive and emotional state of individuals, and thus has an impact on individual behavior. Stimulation is a factor that influences the body’s internal and external situation. It can affect the psychological state or cognitive status of an organism (Lin and Lo, 2016), and then through a series of psychological or cognitive activities, organisms will take an inner and outer reaction to stimuli. The internal reaction is shown by individual attitude, and the external reaction embodied in individual behavior (Lorenzo-Romero et al., 2016). The SOR model is the stimulus-organism-response model. SOR theory, as an important theoretical framework to analyze the response of external stimuli to individual psychology and behavior, constructs the relationship between external environment, individual psychology and behavior. Donovan et al. (1994) applied their improved SOR model to the shopping scene, which promoted the study of SOR model in consumers’ purchase intention and purchase behavior in the Internet era (Djafarova and Bowes, 2021; Shang et al., 2022). In the context of new network media, the “stimulus” in SOR theory is no longer the intuitive objects in the external environment, but through pictures, videos and online interaction to stimulate the user’s consumption psychology, and then produce the consumption impulse and desire that is transferred into actual consumption behavior. Therefore, this study attempts to extend the application of SOR theory to the study of short videos affecting tourist destination decisions.

Specifically, the official promotion short videos’ production quality and the image of tourist destinations they construct will affect the psychological reaction of tourists who then make consumption decisions. When consumers, driven by certain travel purchase motives, purchase tourism products and produce a series of post-purchase behaviors in order to meet certain travel needs, it is a complete travel purchase decision. Therefore, this paper takes the official short videos as the research object and discusses the influence of its stimulus on tourists’ destination decision-making combined with the SOR model.

### Research hypothesis and model construction

**Figure 1:**
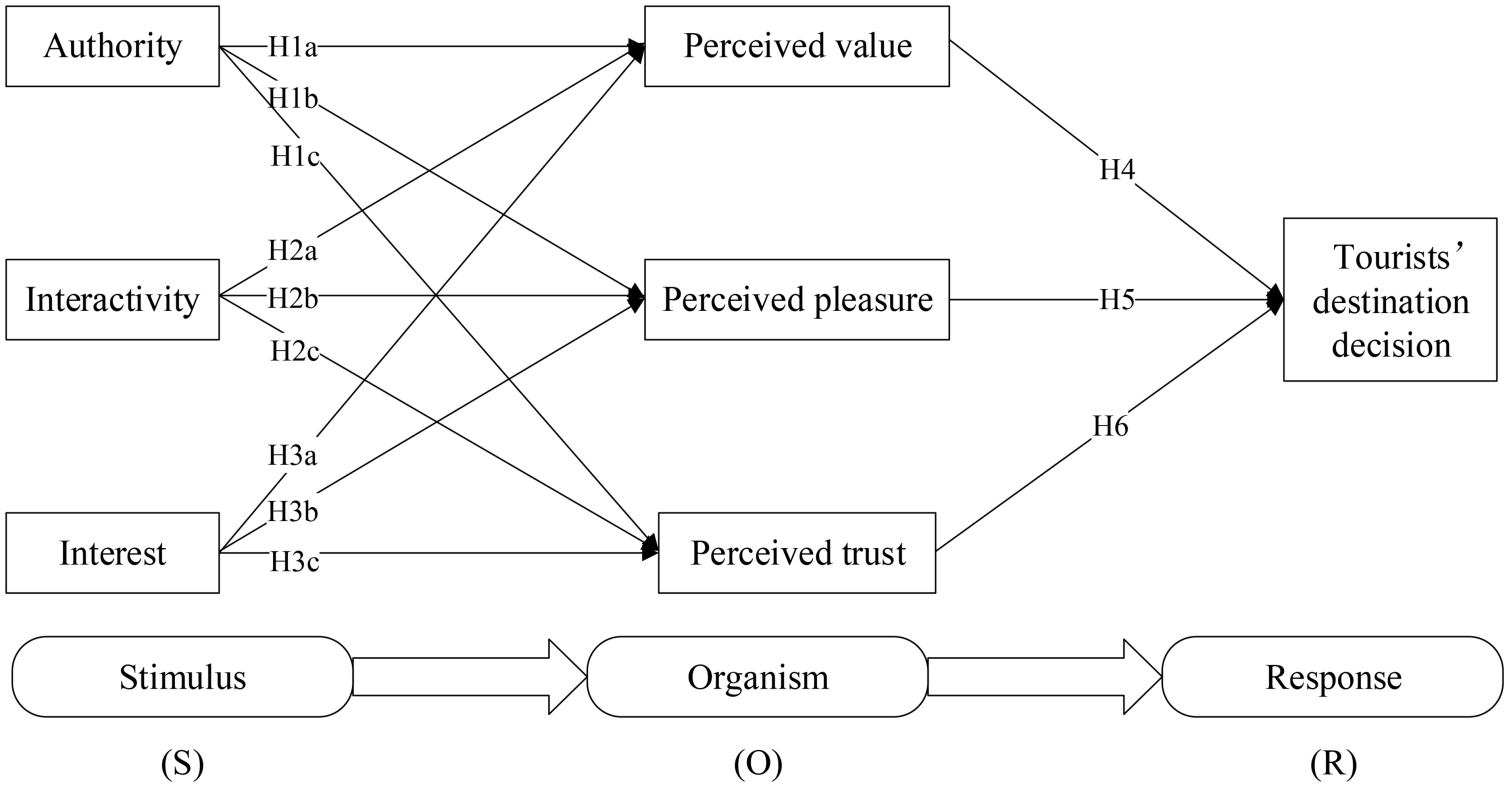
The structural equation model of official promotion short videos’ influence on tourists’ destination decision based on the SOR theory.

Based on the SOR theory, this paper constructs a structural equation model of the influence of official promotion short videos on tourists’ destination decision-making behavior (Figure 1).

The characteristics of official short video accounts and those of short video platforms jointly determine the characteristics of official promotion short videos. In terms of official short video accounts, they have an official background. The government authorizes the official accounts to produce and release official promotion short videos (Yu, 2020). Thus, the information content has official authority and credibility. This is the most distinctive feature that distinguishes official promotion short videos from other short videos. Therefore, we made the hypothesis that the official short video is authoritative. For short video platforms, on the one hand, as a kind of social media, they should meet the users’ interaction and social needs. On the other hand, their operation and development should also meet the entertainment needs of the user group, especially the young group. Hence, the video content should be creative and interesting to make users feel it enjoyable and generate the interest of continuous use after use. Therefore, we made another hypothesis that the official promotion short video is interesting and interactive. To sum up, we hypothesized that official promotion short videos are authoritative, interactive and interesting.

Combined with the SOR model, the official promotion short video, with its special communication mechanism, will be used as an external stimulus to affect the perception or sentiment of potential consumers in the process of watching and then make them produce the decision-making behavior reaction of tendency or avoidance. Taking this model mechanism as a reference, according to the existing research literature results, this study proposes the hypothesis that tourists, as potential consumers, can get stimulation from watching official promotion short videos, which are characteristic of authority, interactivity and interest. After obtaining such an external stimulus, the tourist organism will generate the cognitive reaction of obtaining perceived value, producing perceived pleasure and perceived trust. After the perception reaction is generated, it will further influence the final decision of tourists as potential consumers to accept or reject the destination (i.e., the response). This study verifies and analyzes the above hypotheses based on the questionnaire data.

#### The hypothesis that the official promotion short videos stimulate users

##### Authority

Foucault discussed the relationship between discourse and power in *The Order of Discourse*. In any society, once discourse comes into being, it is immediately controlled, screened, organized, and redistributed by power, and discourse serves power (Grudin, 1996). The important difference between the official promotion short video and the short video released from the we-media or other institutional media lies in the official identity of the account. The new media of the CPC (Communist Party of China) and government organizations that released the official information has high prestige in the public mind. For example, “China Chang’an Net” is the Douyin account of the Political and Judiciary Commission under the Central Committee of the Communist Party of China. As the first political and legal government affairs account on the Douyin platform, it has nearly 40 million fans and popular videos with nearly 8 million likes. It fully reflects the influence and authority of the official mainstream media. The credibility of the information source is the factor with the largest weight in the dimension of communication system, which can affect the perceived risk of users (Tang and Lai, 2021). However, watching the official promotion short videos can have a positive impact on the potential consumers’ impression of the travel destination (Shani et al., 2010). Based on this, this paper puts forward the following hypotheses:

*H1a*: Authority positively affects consumers’ perceived value significantly;

*H1b*: Authority positively affects consumers’ perceived pleasure significantly;

*H1c*: Authority positively affects consumers’ perceived trust significantly.

##### Interactivity

The short video breaks the dimension limitation of time and space, has multiple communication channels, and thus highly fits the factors needed for interaction. Interactivity is one of the distinctive features of short video as a new media platform. Users can give likes, favorites, and comments to express their sentiments while watching the short videos. It is worth mentioning that the official short video account can thus have a quick and effective dialogue with users, respond to questions raised by users, enable users to fully understand the destinations, and facilitate tourists’ decision-making to a certain extent. The content of the videos also spreads further through the recognition and sharing between users. Previous studies have shown that when the travel destination has a crisis of trust due to the epidemic, the destination can respond to the negative comments on the network platform in time through the media, strengthen interaction and communication, and use crisis marketing to effectively resolve the crisis and create opportunities for development (Aktan et al., 2022). Based on this, this paper puts forward the following hypotheses:

*H2a*: Interactivity positively affects consumers’ perceived value significantly;

*H2b*: Interactivity positively affects consumers’ perceived pleasure significantly;

*H2c*: Interactivity positively affects consumers’ perceived trust significantly.

##### Interest

The reason why the official short video platform is welcomed by the public is that compared with the traditional official media content, the short videos released by it no longer use serious and formal official words but adopt humanized narrative techniques to share vivid and relaxing stories through short videos. When making official short videos, some creative clips, interesting scripts, pictures, and sound effects are usually used to produce unique sensory effects to attract users to watch and enjoy. Most of the works tend to be colloquial and fast-paced and often use online buzzwords, which cater to the audience’s mentality of pursuing novelty and interest and further narrow the distance between the government and the public (Jia and He, 2019). Therefore, it makes official information communication more relaxing and popularized so that the public can better accept it. The usefulness, interest, resonance and other characteristics of short video content determine that short video content is easier to spread and enhance user stickiness (Fei and Koo, 2020). Based on this, this paper puts forward the following hypotheses:

*H3a*: Interest positively affects consumers’ perceived value significantly;

*H3b*: Interest positively affects consumers’ perceived pleasure significantly;

*H3c*: Interest positively affects consumers’ perceived trust significantly.

#### Hypothesis about the user response to the official promotion short video stimulus

##### Perceived value

After studying the influence of the perceived value of mobile short video on consumers’ purchase intention, scholars have found that consumers’ perceived value of mobile video is an important factor affecting purchase intention, which can have a positive impact on users’ participation behavior (Wan et al., 2014). Especially in the current context of the uncertain epidemic situation, tourists cannot experience tourism resources offline and thus need to make decisions after weighing the perceived value. In the face of short video advertising and marketing, users do not blindly follow but actively choose. The core competitiveness and brand personality of short video account have a significant impact on users’ perceived value (Zeng, 2021). Only when tourists perceive that the consumption behavior is meaningful and valuable can they turn their potential purchase intention into practical actions. The purpose of tourist decision-making is to maximize tourism utility. Before making a destination decision, consumers’ perceived value evaluation and psychological feelings in destination promotion videos may affect consumers’ final consumption behavior. Consumers’ perceived value has a positive impact on consumer happiness and stickiness (Ren et al., 2021). Based on this, this paper puts forward the following hypothesis:

*H4*: Perceived value has a positive and significant impact on tourists’ destination decisions.

##### Perceived pleasure

Individuals’ sentimental energy will be generated in an interactive ceremony, which is deep and strong, and will affect users’ next behavior. Studies have shown that sentimental response and product purchase intention also have a significant positive impact (Li, 2019). In the process of watching the official promotion short videos, the users, as potential consumers, may be stimulated by the audio-visual effects of the videos and then gain inner pleasure, thus generating yearning for destinations and affecting their decision-making of the destinations. Based on this, this paper puts forward the following hypothesis:

*H5*: Perceived pleasure has a significant positive impact on tourists’ destination decisions.

##### Perceived trust

The COVID-19 pandemic has seriously affected people’ willingness to travel (Hao et al., 2021). After the outbreak of the pandemic, Chinese nationals reduced their preferences in all travel modes and most of the travel forms (Huang et al., 2021). Influenced by the uncertainties in the current epidemic situation, the coronavirus has not been thoroughly controlled, so the risk of coronavirus contagion in tourist destinations will increase due to the gathering of people. Therefore, tourists are worried and uneasy about travel universally, and will be more anxious in the absence of official authoritative information. COVID-19 was shown to negatively affect the travel intention (Cho, 2021). Tourists’ travel decisions are largely based on their own risk assessment and safety perception of the epidemic situation. Destination crisis marketing should provide positive information about destinations to ease their risk perception and enhance their trust. At this point, the government’s emergency management department should use various media to intensify epidemic information release and publicize the epidemic prevention and control measures in the tourist destinations. These measures may give tourists the right to know, eliminate their tension and anxiety caused by the information asymmetries. In this way, the government can reduce tourists’ perception of the risk and enhance their trust in government’s authority. Previous studies (e.g., Avraham, 2015; Assaker and O’Connor, 2020) have shown that when a destination is hit by a crisis such as a terrorist attack or a natural disaster, the emergency management department (DEM) can reduce tourists’ risk perception and generate a sense of trust. Based on this, this paper puts forward the following hypothesis:

*H6*: Perceived trust has a significant positive impact on tourists’ destination decisions.

## Data collection and analysis

### Variable measure

Based on the existing research results, this paper designed a variable measurement scale and then designed a questionnaire according to the items of each variable and the theme of this study. All the measures in the questionnaire adopted the five-level Likert scale. Seven variables were selected in this study. The basis for variable selection and questionnaire design items are shown in Table 1.

**Table 1:** Variable screening and basis.

Variable	Number	Item	Source of reference
Authority	Q9	Compared with other accounts, the official account is a highly professional expert.	Zhang et al. (2010), Guo et al. (2019)
	Q10	Compared with other accounts, the official account is certified and verifiable by the platform.	
	Q11	Compared with other accounts, the official account is reliable and trustworthy.	
	Q12	Compared with other accounts, the official account is generally recognized by the public.	
Interactivity	Q13	Through the official account, I can search, consult, comment, share and collect all kinds of travel information.	Coursaris et al. (2012), Yuan and Wang (2015)
	Q14	Through the official account, I can communicate with the tourism authorities and make suggestions.	
	Q15	Through the official account, my questions can be answered in time.	
	Q16	Through the official account, I like to participate in the interactive communication of the official promotion short videos.	
Interest	Q17	I think the official promotion short videos are interesting	Jin (2015),
	Q18	The interesting content is an important reason why I pay attention to this kind of short video.	Shen and Zhao (2018)
	Q19	Whether the official video is wonderful and interesting will affect my subsequent decision.	
Perceived value	Q20	By watching the official promotion short video of the travel destinations, I can know the tourism resources of the destinations.	Sweeney and Soutar (2001), Wang et al. (2004)
	Q21	By watching the official promotion short video of the travel destination, I can judge whether it can meet my travel needs.	
	Q22	The travel information provided in the official promotion short video was helpful to me.	
Perceived pleasure	Q23	It was enjoyable to watch the official promotion short video	Hassanein and Head (2007)
	Q24	After watching the official short video, I feel relaxed.	
	Q25	After watching the official short video, I feel happy.	
Perceived trust	Q26	After watching the official short video, it will be safer to travel to the destination.	Stone and Grønhaug (1993), Han (2005)
	Q27	After watching the official short video, it will not make me nervous if I travel to the destination.	
	Q28	After watching the official short video, it will not make me worried if I travel to the destination.	
Tourist destination decision-making	Q29	Before the actual travel, I will search the official promotion short video for reference.	Bansal and Voyer (2000), Park et al. (2007)
	Q30	The travel information in the official promotion short video has changed my attitude and opinion towards a certain travel destination.	
	Q31	The travel information in the official promotion short video will have a direct impact on my decision on a travel destination.	
	Q32	The travel information in the official promotion short video will help me make the decision about the destination.	

### Sample descriptive statistics

We distributed a total of 435 questionnaires to Chinese Internet users *via* an online questionnaire platform in China. Among them, the questionnaires that said they had not watched the official promotion videos, that had identical answer options throughout, and that were completed in less than 1 min were deleted. The remaining valid questionnaires were 397, with an effective rate of 91.3%. It indicates that these respondents all have had the real experience of watching official promotion short videos before they participated in the survey. 51% of the respondents were female, and 49% were male. The education level is concentrated in junior college to an undergraduate degree, accounting for 77.7%. The age is concentrated in 21–40 years old, accounting for 70.2%. Among the ways to get information about the destination, social media and asking relatives and friends are the most popular ways. In the past 3 years, about 85% of the respondents have traveled during the epidemic situation, indicating that all the respondents have certain travel experiences during the epidemic situation.

### Reliability and validity test

Through SPSS25 data processing and reliability analysis, the overall standardized Cronbach ‘α coefficient of the questionnaire is 0.963, which shows that the questionnaire has good internal consistency and stability. The standard loads are all greater than 0.7, which shows that the observed variables explain the latent variables well. KMO = 0.974, indicating that the correlation between variables is strong and suitable for factor analysis. The combined reliability of Cronbach ‘α and CR of each latent variable is greater than 0.7, which shows that the internal consistency is good and it has good reliability and reliability.

The validity test is to verify the validity of the questionnaire, which is divided into aggregation validity and discriminant validity. Aggregation validity is measured by the AVE value. When AVE is greater than 0.5, the aggregation validity of the scale is good, and all latent variables AVE of this questionnaire are greater than 0.5. Discriminant validity is measured by comparing the square root of latent variable AVE with the correlation coefficient between variables. As shown in Table 2, the diagonal is the square root of each latent variable AVE, and the rest is the correlation coefficient. The square root of each latent variable AVE in the table is greater than the correlation coefficient, which shows that the sample has good discrimination validity.

**Table 2:** Analysis of the model fitting degree.

Indicators	*X*^2^/*df*	NFI	RFI	IFI	CFI	RMSEA
Value	2.802	0.981	0.965	0.988	0.988	0.064
Reference range	<3	>0.8	>0.8	>0.8	>0.8	<0.08

### Hypothesis testing

According to the hypothesis of this research model, the structural equation model is constructed by AMOS24 software, and six adaptation indexes, *x*^2^/*df*, NFI, RFI, IFI, CFI, and RMSEA, are selected to evaluate the fitting degree of the structural equation. It is generally believed that when the ratio of chi-square degrees of freedom is <3, GFI, NFI, and IFI are close to 1, and RMSEA is <0.08, the fitting degree of the model is better (Table 2).

**Figure 2:**
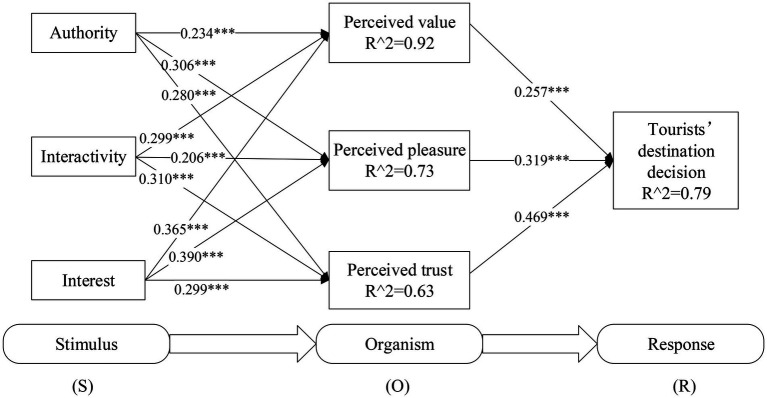
Research model path coefficient and its significance level. *Means *p* < 0.05, **means *p* < 0.01, ***means *p* < 0.001.

The significance level of each variable was calculated by Boot Strapping, and the stability of data results was ensured by 2000 samples. When *T* > 1.96, it means reaching a significant level of 0.05; when *T* > 2.58, it means that it reaches the significant level of 0.01; when *T* > 3.29, it means that it reaches a significant level of 0.001. Path analysis and significance level are shown in the figure. *R*^2^ represents the degree to which the explanatory variable explains the explained variable. The analysis results are shown in Figure 2 and Table 3.

**Table 3:** Research results.

Research hypothesis	Path correlation	Path coefficient	*T* value	Significance level	Hypothesis test results
H1a	Authority → Perceived value	0.234	5.226	***	Support
H1b	Authority → Perceived pleasure	0.306	6.913	***	Support
H1c	Authority → Perceived trust	0.280	6.084	***	Support
H2a	Interactivity → Perceived value	0.299	6.478	***	Support
H2b	Interactivity → Perceived pleasure	0.206	4.525	***	Support
H2c	Interactivity → Perceived trust	0.310	6.550	***	Support
H3a	Interest → Perceived value	0.365	7.073	***	Support
H3b	Interest → Perceived pleasure	0.390	7.633	***	Support
H3c	Interest → Perceived trust	0.299	5.620	***	Support
H4	Perceived value → Tourists’ destination decision	0.257	5.887	***	Support
H5	Perceived pleasure → Tourists’ destination decision	0.319	7.160	***	Support
H6	Perceived trust → Tourists’ destination decision	0.469	10.856	***	Support

## Results

### Official short video stimulates the user significantly and gets a positive response

Authority positively affects perceived value, perceived pleasure, and perceived trust significantly. Official short video media has strong authority because of its position of official certification, providing authentic and reliable information, owning complete and efficient internal resources, and ensuring the rapidity and credibility of its information to a certain extent (Yu, 2020). In the process of searching for and receiving information, users tend to prefer credible information sources, so the authority can enhance users’ value perception and reduce risk perception (Tang and Lai, 2021). Therefore, when watching the official promotion short videos, the public often has a high acceptance of the image of tourist destinations and tourism resources displayed, which stimulates their perception and sentimental response positively. At the same time, high-quality information and publicity ability, linked with offline tourism work, enable users to give feedback and enhance the authority of the government after getting a good travel experience.

Interaction positively affects perceived value, perceived pleasure, and perceived trust significantly. The interactive mechanism of official promotion short videos includes one-way behaviors such as users’ likes, comments, and sharing and two-way behaviors such as answering questions in private letters. Interactive behavior can generate group recognition (Liu, 2021) and improve perception and sentimental stimulation in recognition. The more frequent and extensive the interaction covers, the more complete the destination image can be shaped. At the same time, the recommendation mechanism of the platform will also generate feedback and achieve positive growth—bring perceived value, gain perceived pleasure and enhance perceived trust in the interaction. The interaction will also deepen users’ cognitive impression of concerns, form a cognitive network of tourist destination image, and shape a good perception of urban tourism image.

Interest affects perceived value, perceived pleasure, and perceived trust significantly. The reason why short videos can get great attention and online traffic in the new Internet era cannot be separated from their own interesting and entertaining characteristics. Interesting and creative short videos can catch users’ eyes in a short time and bring high-quality perception enjoyment to users, so that they can fully understand relevant information and generate positive sentiments in just a few tens of seconds. Interesting videos can highlight marketing priorities and deepen users’ perceptions. Short videos of “magic brainwashing” are more likely to occupy users’ minds. The more interesting they are, the easier for tourists to obtain an immersive experience and relaxed and pleasant mood, so as to meet their various needs for the destination, eliminate their anxiety, and generate consumption impulses or plans.

### User’s reaction positively affects their destination decision-making behavior significantly

Perceived value has a significant positive impact on tourists’ destination decision-making. In the process of watching the official promotion short videos, users can get to know the tourism resources, policies, epidemic prevention and control measures and other conditions in this area, get stimulated by the information obtained and compare the requirements of travel destinations accordingly. If they meet the needs, the users will have travel willingness and their online purchase decision will further be influenced by the reference price and online comments (Li et al., 2017).

Perceived pleasure has a significant positive impact on tourists’ destination decision-making. When watching the official promotion short videos, if the users obtain a relaxed and pleasant sentimental reaction, it can not only improve their good feelings towards the tourist destination and the expectation of the destination tourism resources, but also stimulate them to respond positively, thus generating a strong willingness and desire to travel. Especially in the livestreaming e-commerce platform, consumers’ perceived pleasure and perceived trust together significantly affect their purchasing behavior (Liu et al., 2020).

Perceived trust has a significant positive impact on tourists’ destination decision-making. Under the background of the normalization of epidemic prevention and control in China, users are eager to travel on the one hand and worried and nervous about potential safety risks in the process of travel on the other hand, thus affecting their travel decisions. Consumer’s trust is the first key factor affecting their behavior choice (Yang et al., 2016). By releasing the official promotion short videos, local governments publicize reliable information about tourism resources and display with the authoritative and official image the real situation of epidemic prevention and control in the travel destination. It can dispel users’ worries and anxiety and alleviate their risk perception of the place to a certain extent, thereby increasing their willingness to travel and influencing their subsequent decision-making behavior.

## Discussion

Based on the above research results, the study holds that official promotion short videos can significantly influence tourists’ destination decisions. Accordingly, the following three aspects can be used to enhance the publicity and promotion capacity of local governments’ official short videos.

Firstly, improve the authority of official short videos and strengthen the credibility of the official account. Authority is one of the remarkable features that differentiate the official promotion short video from other tourism promotion short videos, and it is also one of the important ways to enhance users’ perceived trust. Due to the uncertainties of COVID-19 epidemic development, in order to enhance users’ perception and trust, it is necessary to strengthen the credibility of the official short video account. The production and expression of official short videos should be normative, and a balance should be sought between official discourse and mass media, and the essential characteristics of seriousness and authority should not be lost in catering to netizens blindly. In the production and release of official short videos, it is necessary to ensure the authenticity and reliability of the information, edit and proofread the video carefully, and avoid false publicity and exaggerated marketing. Keep the quality while obtaining online traffic, so as to create a long-term reputation and good positive feedback. Therefore, the local government should strengthen the guidance, supervision, and content review of the official short video content, prevent the dissemination of vulgar content or that worsens the image of tourist destinations. Meanwhile, prevent it from being blindly casual, pay attention to balancing publicity and entertainment, create a healthy publicity environment, and maintain the credibility of the government media account.

Secondly, strengthen the official short video interaction mechanism and expand the influence of the official account. For short video marketing, interactive mode is an important strategy of short video marketing, which forms a cycle from watching video to paying attention to anchors to shopping through the interaction between people and commodities (Qian, 2021). For the official government affairs account, interaction is an important means of communication between the government and the public. The benign interaction mechanism through likes, comments, sharing and other behaviors can not only stimulate the interactive users’ perception, sentiment and trust, but also bring exposure and communication power to the account, so as to improve the efficiency of publicity. The official promotion short video subjects should further strengthen the interactive mechanism, build a bridge of communication between the official and netizens, shorten the psychological distance between each other, enhance tourists’ recognition, develop through real-time responses and convenient services high-quality interactive channels for the public, and shape a good government image. Specifically, we can make full use of the special communication mechanism of the short video platform—theme posting online—to set agenda topics actively. That is, the official account can launch topic labeling under characteristic scenic spots or activities, actively guide tourists to post and record the travel experience online, and invite influential network key opinion leaders to experience personally. In this way, it may help generate perceptual interaction, gain sensory experience and obtain emotional resonance, and create attractive “online celebrity scenic spots” or “online celebrity projects.” In addition, tourists’ feedback and personal evaluation are also important factors in improving the image of tourist destinations. Paying attention to positive and satisfactory feedback and understanding and responding to negative feedback can better dispel tourists’ negative sentiments and improve the good feelings and trust of other tourists.

At last, stimulate the creativity of official short video content and enhance the enjoyment of the official short videos. The rapid popularity of short videos on the Internet platform is due to their eye-catching creativity and funny entertainment creation forms. With entertaining and creative language and content, the official short video improves the original blunt way of communication and makes users feel the kindness and interest of the official media. On the one hand, local governments should strengthen the top-level design, rely on the overall development strategy of the city, and sort its overall atmosphere, traditional culture, public resources, scenic spots, etc. That is, they should clarify the advantages of the tourism resources, position the characteristics of the city accurately, and enhance the users’ experience of watching the promotion short videos. On the other hand, official accounts should also pay attention to the shooting quality and editing level of short videos, deeply explore local characteristics, create original videos, adopt diversified content production methods, strengthen brand communication and brand image, and improve users’ perceived value (Cui et al., 2019). The official accounts can use the popular narrative method preferred by the public to provide them with an immersive experience that delivers the sense of presence (Cao et al., 2021). The local governments can also develop and integrate their own traditional food, entertainment activities, civil life, urban music, and other elements and realize the value and moral guidance through short videos, so as to highlight a good city image and stimulate tourists’ desire to travel.

## Conclusion

Based on the SOR theory and structural equation model, this study used a questionnaire as an empirical analysis method to explore the influence factors of official promotion short videos on tourists’ destination decisions in the post-epidemic era. The research results show that the tourism promotion short videos released by government departments’ official accounts on Douyin short video platform have three characteristics: authoritative, interactive, and interesting. Furthermore, based on the tourists’ trust in the government, they are significantly stimulated after watching the official promotion short videos, which positively affect their destination decision-making behavior. Theoretically, the study contributes to expand the application scope of SOR theory from traditional consumer purchasing behavior to tourist destination decisions, which not only enriches the application scope of the theory, but also provides a new theoretical perspective for the research of tourist destination decision. Given the uncertain situation of the epidemic, this study has a positive meaning for local governments to use new media platforms to publicize and promote tourism resources and restore tourism and the local economy.

The limitation of this study is mainly reflected in the quantity of questionnaire data which is still insufficient, and the model’s validity needs to be further tested. To solve this problem, the researchers plan to conduct field investigations in selected places that benefit from the official promotion short videos, and obtain first-hand data by interviewing tourists who have watched the promotion short videos and travel to the destinations, so as to improve the effectiveness of the model.

## Data availability statement

The raw data supporting the conclusions of this article will be made available by the authors, without undue reservation.

## Author contributions

JJ: propose the topic and research design and paper revision, YH: write the first draft of the paper and participate in the revision of the paper. WL: participated in the design of the questionnaire and analyzed the questionnaire data. DL: participate in paper revision. All authors contributed to the article and approved the submitted version.

## Funding

This paper was supported by the National Social Science Foundation of China (Grant number: 17CGL074).

## Conflict of interest

The authors declare that the research was conducted in the absence of any commercial or financial relationships that could be construed as a potential conflict of interest.The reviewer MG declared a shared affiliation with the author DL to the handling editor at the time of review.

## Publisher’s note

All claims expressed in this article are solely those of the authors and do not necessarily represent those of their affiliated organizations, or those of the publisher, the editors and the reviewers. Any product that may be evaluated in this article, or claim that may be made by its manufacturer, is not guaranteed or endorsed by the publisher.

## Footnotes

^1^https://www.thepaper.cn/newsDetail_forward_12213569贺娇龙、刘洪走红网络背后:基层文旅干部正寻找产业突围之路 (Behind the Internet fame of HE Jiaolong and LIU Hong: grassroots leaders in cultural and tourism departments are looking for a way to break through).
